# Superplot3d: an open source GUI tool for 3d trajectory visualisation and elementary processing

**DOI:** 10.1186/1751-0473-8-19

**Published:** 2013-09-30

**Authors:** Luke J Whitehorn, Frances M Hawkes, Ian AN Dublon

**Affiliations:** 1 Eurisko Studios, Canterbury Street, Gillingham ME7 5XL UK; 2 Natural Resources Institute, University of Greenwich, Chatham Maritime, Kent ME4 4TB UK; 3 Unit of Chemical Ecology, Department of Plant Protection Biology, Swedish University of Agricultural Sciences, Växtskyddsvägen 3, PO Box 102, SE-230 53 Alnarp , Sweden

**Keywords:** Trajectory, Matlab, Gui, 3d, Framework

## Abstract

When acquiring simple three-dimensional (3d) trajectory data it is common to accumulate large coordinate data sets. In order to examine integrity and consistency of object tracking, it is often necessary to rapidly visualise these data. Ordinarily, to achieve this the user must either execute 3d plotting functions in a numerical computing environment or manually inspect data in two dimensions, plotting each individual axis.

Superplot3d is an open source MATLAB script which takes tab delineated Cartesian data points in the form *x*, *y*, *z* and *time* and generates an instant visualization of the object’s trajectory in free-rotational three dimensions. Whole trajectories may be instantly presented, allowing for rapid inspection. Executable from the MATLAB command line (or deployable as a compiled standalone application) superplot3d also provides simple GUI controls to obtain rudimentary trajectory information, allow specific visualization of trajectory sections and perform elementary processing.

Superplot3d thus provides a framework for non-programmers and programmers alike, to recreate recently acquired 3d object trajectories in rotatable 3d space. It is intended, via the use of a preference driven menu to be flexible and work with output from multiple tracking software systems. Source code and accompanying GUIDE .fig files are provided for deployment and further development.

## Findings

Comparatively recent developments in object trajectory capture allow easy acquisition of three-dimensional (3d) flight data from moving objects by capturing output from two fixed position cameras with overlapping *x* and *y* axes and applying on-the-fly conversion to Cartesian *xyz* co-ordinates [1-6]. One such commercially available tracking solution is Trackit 3D (SciTrackS, GmbH) [3]. Trackit 3D allows the trajectories of very small objects to be tracked, including those of very small insects [3,7,8].

In an ordinary tracking event using a standard PAL analogue camera at 25 frames s^-1^, Trackit 3D utilises the 2 fields contained within each frame. Irrespective of whether tracking is correct, one second of recorded output equates to 50 textual lines of data with each line intended to represent the current position of the object being tracked. Acquisition of flight data by this method rapidly generates vast sets of Cartesian output that necessitates the use of 3d visualisation in order to make it meaningful and ensure tracking integrity remains consistent. For example, in a real world scenario, 22 minutes of insect tracking will create output that exceeds the bounds of an .xls file (65,536 rows). Whilst it is easy to interrogate such data with the *plot3* command in MATLAB (The Mathworks, MA) or a free equivalent numerical computing environment such as GNU Octave [9], doing so provides very basic output and necessitates that the user apply specific syntax in order to format or interrogate plotted output or alter which points are plotted. Whilst this may be suitable for a short trajectory, it becomes increasingly arduous when the track duration is over several minutes or indeed if only a few seconds of the track are of interest. In addition, further analysis in such an environment requires considerable familiarisation with MATLAB or Octave.

**Figure 1:**
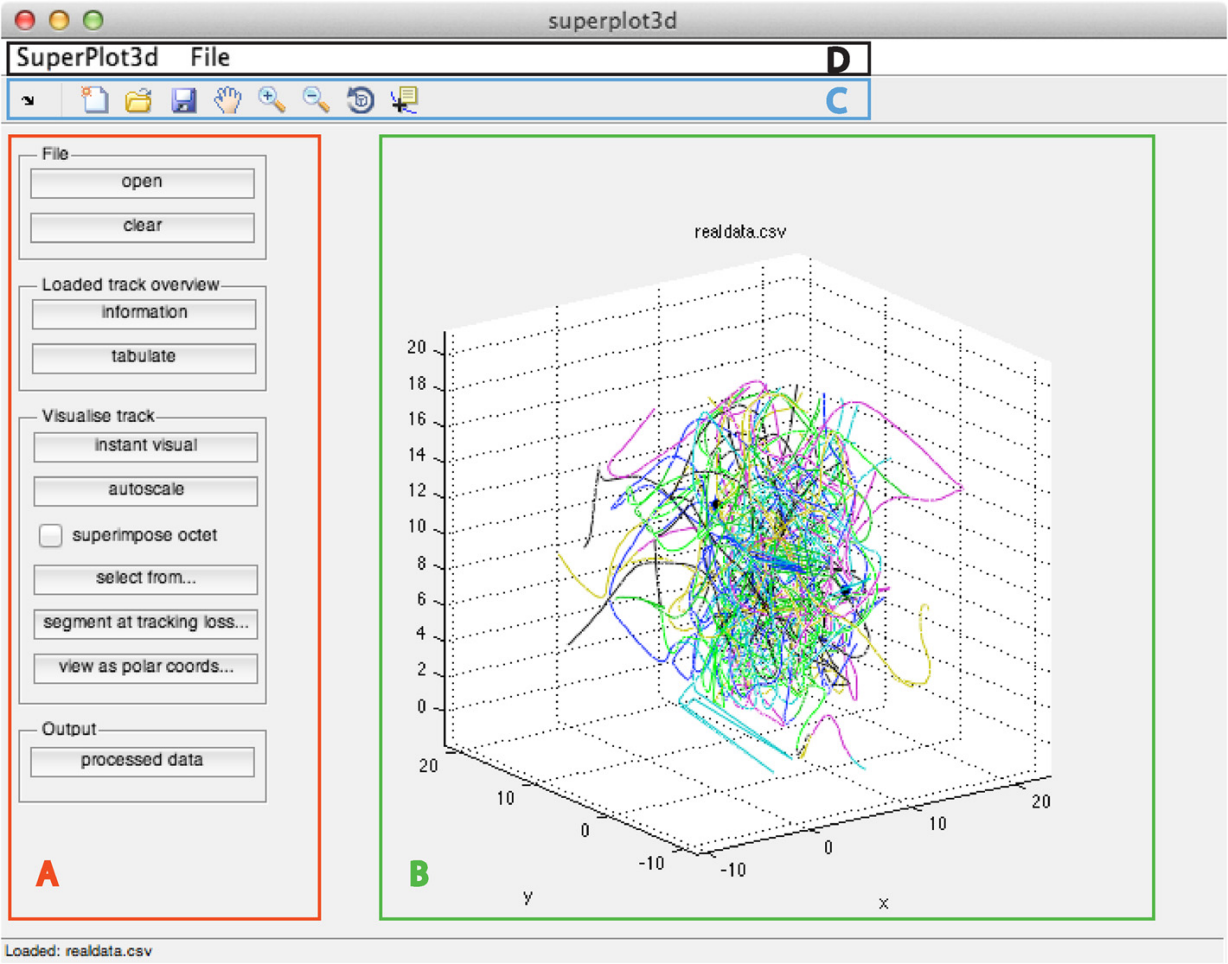
**Interface elements.** To the left are a series of buttons instigating sub-functions (Outline **A**). The dataset may be viewed on the main plot figure axis (Outline **B**). View controls are located in the menubar (Outline **C**) and a global menu exists within the window (Outline **D**).

**Figure 2:**
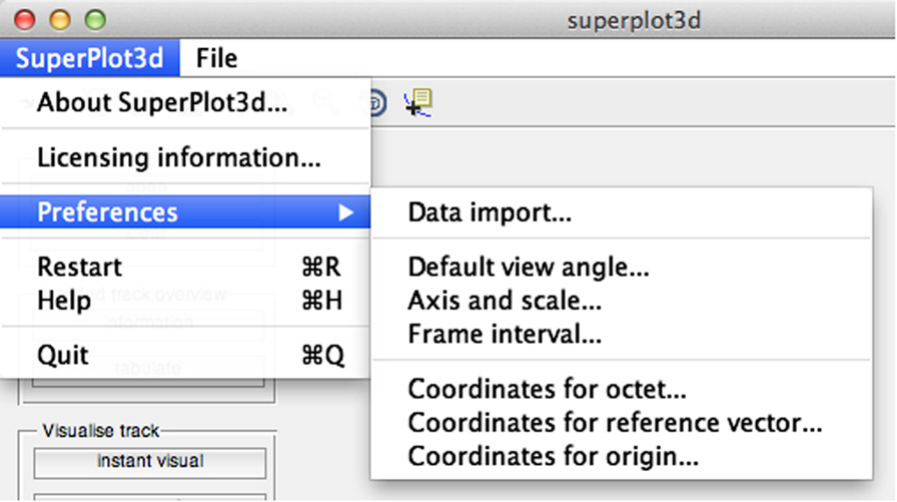
Superplot3d menu allowing configuration of preferences.

**Figure 3:**
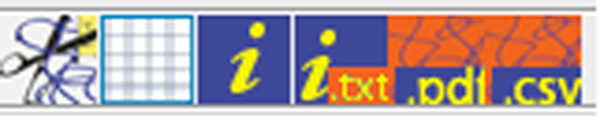
**Menubar buttons appearing on child windows.** Here showing: Cut, Tabulate, Get info, Export info as .txt, Export graphic as .pdf, Export dataset as .csv.

**Figure 4:**
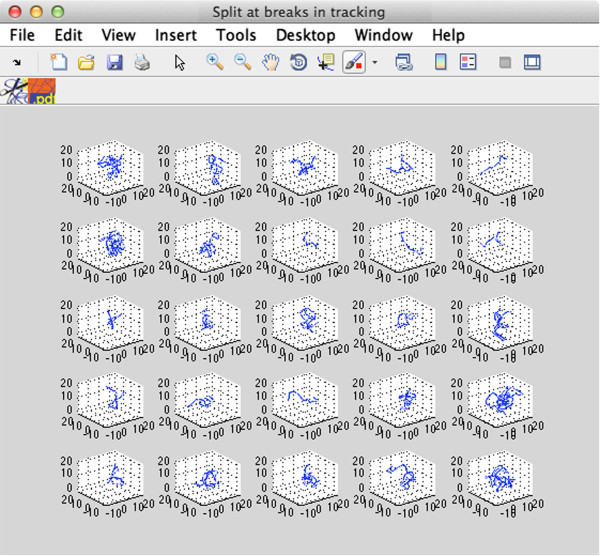
The first 50 parts of a dataset, automatically split into constituent parts.

Superplot3d allows a user with no previous MATLAB experience to instantly inspect large datasets of flight trajectory where data is provided as *x*, *y*, *z* and *time*. The software uses MATLAB’s underlying painters rendering engine along with calls to Matlab graphical user interface functions to output the complete object trajectory, or subsets of it. Trajectory view is auto-scalable or user definable using menu-driven preferences. The user is able to free-rotate the trajectory in three dimensions, select key sections to view and convert regions from Cartesian to cylindrical polar coordinates for further analysis. The speed, idiothetic 3d turning angle and vector reference heading of the tracked object can also be derived. In addition, it is easy to split the trajectory at the point at which an interruption to the tracking data occurs, producing multiple subsets of a tracked trajectory. This enables the user to rapidly gauge the value of the data as a whole, and of its constituent parts. See Figures 1, 2, 3, 4 for an overview.

Furthermore, inclusion of the source code and accompanying GUIDE .fig file allow the user to readily modify this program as necessary.

### Methods and implementation

Superplot3d is a platform independent MATLAB *.m* file (available under creative commons license from http://superplot3d.slu.se) compatible with MATLAB R2010a (R14) and above. Moreover, if the MATLAB compiler toolkit is present on the development machine, it is deployable as a standalone executable. This allows a small self-contained application to be built enabling users with the appropriate MATLAB Compiler Runtime (MCR) installed on their system to use all functions without having a MATLAB installation present. Any built application is thus designed to neatly co-exist with the track acquisition software on the data acquisition workstation, allowing for near instantaneous trajectory inspection.

### Implementation

Data provided as tab delimited, or comma separated files may be read directly into memory. Data are error checked for obvious anomalies (mismatched matrix dimensions) and prolonged periods of tracking loss are truncated to one line between tracks. Said data are passed to a process function, which after being indexed by user defined variables (defined in the preference menu), generates a structure containing processed data output. Whole data may be visualised with a press of a button. In addition, tracking may be viewed from subsequent user defined points, the entire set of tracks may be split up at breaks in tracking where one or more *NaN* is returned, the entire dataset can be viewed as cylindrical polar coordinates and the user may be able to select specific tracks, 'cutting’ them, accordingly. Outputted data may be returned as a .csv file containing columns containing *x*, *y*, *z*, *t*, distance between tracked point 1 and tracked point 2, distance between tracked point 1 and tracked point 3, speed, idiothetic turning angle and 3d angle in relation to a definable reference vector. Calculations are illustrated in Table 1.

**Table 1:** Calculations for track analysis

**Track parameter**	**Description**	**Calculation**	**Unit**
dist1	Distance between first point pair (point *i* and *i*-1)	*P*_*i*_ - *P*_*i* - 1_	Dependent upon original data
dist2	Same as above for *i*+1 and *i*		Same as above
Speed	Distance between point pair divided by time difference	$\frac{\left|P_{i}-P_{i-1}\right|}{t_{i}-t_{i-1}}$	Distance unit time unit ^-1^
Idiothetic turning angle	Angle of 2nd point pair, relative to first point pair	$\theta_{i}=\text{acos}\left(\left(\frac{P_{i+1}-P_{i}}{\left|P_{i+1}-P_{i}\right|}\right)\cdot \left(\frac{P_{i}-P_{i-1}}{\left|P_{i}-P_{i-1}\right|}\right)\right)$	°
Three-dimensional angle	Angle, relative to reference vector	$\theta_{i}=\text{acos}\left(\left(\frac{P_{i}-P_{i-1}}{\left|P_{i}-P_{i-1}\right|}\right)\cdot w\right)$ ***w ***= reference vector	°

## Conclusions

Superplot3d is a framework for rapidly evaluating object trajectory data. It is designed to form a platform for further development and in itself provides a useful tool for trajectory examination. It has been used for real world mosquito flight trajectory data at the Gibson laboratory, Natural Resources Institute, University of Greenwich UK.

## Competing interests

The authors declare that they have no competing interests.

## Authors’ contributions

IAND and LW developed the software from IAND’s initial code. IAND, FMH and LW wrote the manuscript. All authors have read and approved the final manuscript.

## Authors’ information

IAND is a postdoctoral research fellow in chemical ecology at the Swedish University Of Agricultural Sciences, SLU Alnarp. LW is a visual effects artist and physics graduate. FMH is a postdoctoral research fellow at the University of Greenwich.
